# Clinical Results of a Massive Blood Transfusion Protocol for Postpartum Hemorrhage in a University Hospital in Japan: A Retrospective Study

**DOI:** 10.3390/medicina57090983

**Published:** 2021-09-18

**Authors:** Daigo Ochiai, Yushi Abe, Rie Yamazaki, Tomoe Uemura, Ayako Toriumi, Hiroko Matsuhashi, Yuya Tanaka, Satoru Ikenoue, Yoshifumi Kasuga, Ryuji Tanosaki, Mamoru Tanaka

**Affiliations:** 1 Department of Obstetrics and Gynecology, Keio University School of Medicine, Tokyo 160-8582, Japan; y.abe@keio.jp (Y.A.); tanakayuya0108@yahoo.co.jp (Y.T.); sikenoue.a3@keio.jp (S.I.); 17yoshi23.k@gmail.com (Y.K.); mtanaka@keio.jp (M.T.); 2 Center for Transfusion Medicine and Cell Therapy, Keio University School of Medicine, Tokyo 160-8582, Japan; r-yamazaki@keio.jp (R.Y.); tomoe.uemura@adst.keio.ac.jp (T.U.); ayako.toriumi@adst.keio.ac.jp (A.T.); hiroko.matsuhashi@adst.keio.ac.jp (H.M.); rtanosak@keio.jp (R.T.)

**Keywords:** postpartum hemorrhage, massive blood transfusion protocol, delivery

## Abstract

*Background and objectives:* Massive postpartum hemorrhage (PPH) is the most common cause of maternal death worldwide. A massive transfusion protocol (MTP) may be used to provide significant benefits in the management of PPH; however, only a limited number of hospitals use MTP protocol to manage massive obstetric hemorrhages, especially in Japan. This study aimed to assess the clinical outcomes in patients in whom MTP was activated in our hospital. *Materials and Methods:* We retrospectively reviewed the etiology of PPH, transfusion outcomes, and laboratory findings among the patients treated with MTP after delivery in our hospital. *Results:* MTP was applied in 24 cases (0.7% of deliveries). Among them, MTP was activated within 2 h of delivery in 15 patients (62.5%). The median estimated blood loss was 5017 mL. Additional procedures to control bleeding were performed in 19 cases, including transarterial embolization (18 cases, 75%) and hysterectomy (1 case, 4.2%). The mean number of units of red blood cells, fresh frozen plasma, and platelets were 17.9, 20.2, and 20.4 units, respectively. The correlation coefficients of any two items among red blood cells, fresh frozen plasma, platelets, blood loss, and obstetrical disseminated intravascular coagulation score ranged from 0.757 to 0.892, indicating high levels of correlation coefficients. Although prothrombin time and activated partial thromboplastin time levels were significantly higher in the <150 mg/dL fibrinogen group than in the ≥150 mg/dL fibrinogen group at the onset of PPH, the amount of blood loss and blood transfusion were comparable between the two groups. *Conclusions:* Our MTP provides early access to blood products for patients experiencing severe PPH and could contribute to improving maternal outcomes after resuscitation in our hospital. Our study suggests the implementation of a hospital-specific MTP protocol to improve the supply and utilization of blood products to physicians managing major obstetric hemorrhage.

## 1. Introduction

Massive postpartum hemorrhage (PPH) is the most common cause of maternal death worldwide [1,2]. Life-threatening massive PPH occurs in one out of every 300 cases [3], and its onset is difficult to predict before delivery. Once it occurs, it can result in nonstandard requests for blood products without any protocol guiding the administration of a large amount of blood in a short period.

Massive blood transfusion is traditionally defined as transfusion of 10 or more units of packed red blood cells (RBCs) in less than 24 h [4,5,6]. Massive transfusion in obstetrics requires extensive collaboration between the obstetrics, anesthesiology, and transfusion teams and consumes a large amount of blood in hospitals with limited blood product storage [7]. The tasks in these cases are demanding such that individual clinical expertise is often insufficient, and teams from obstetrics, operating rooms, and transfusion departments often do not work well together. Therefore, facilities must be prepared in providing effective treatment in such emergencies. The clinical team is expected to develop, implement, and regularly review obstetric bleeding protocols [1,4,5,6].

Many hospitals with accredited trauma centers have adopted a massive transfusion protocol (MTP) [8,9]; however, only a limited number of hospitals use the MTP protocol to manage massive obstetric hemorrhages, especially in Japan. Recently, we established an obstetric MTP suitable for our hospital to ensure a rapid and optimal supply of blood products in the acute phase for the resuscitation of patients with uncontrolled PPH.

This observational study aimed to assess the etiology of obstetric hemorrhage, transfusion requirements, and clinical outcomes in patients in whom MTP was activated in our hospital.

## 2. Materials and Methods

A database review was conducted to identify the patients who were treated with the MTP after delivery at Keio University Hospital between 1 October 2015 and 31 July 2021. Owing to the retrospective design of this study, an opt-out consent was obtained. The study was approved by the Research Ethics Review Board of Keio University (no. 20150103).

**Figure 1:**
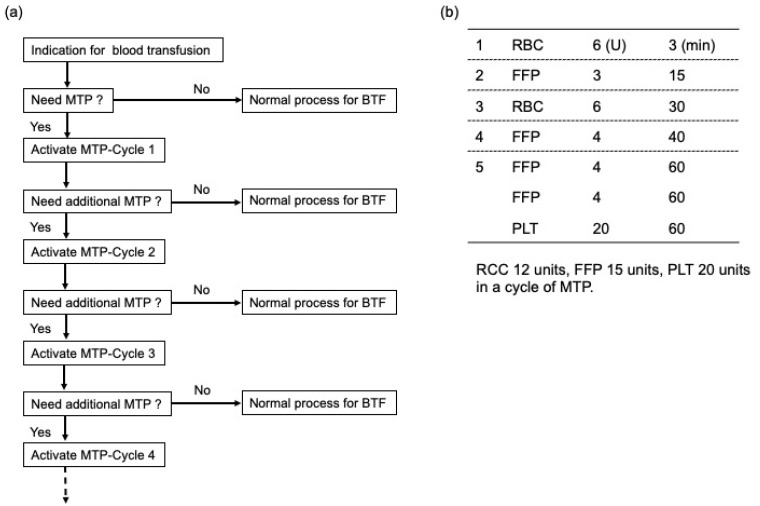
Flow diagram illustrating the massive blood transfusion protocol. (**a**) Flow diagram of massive blood transfusion protocol. (**b**) Blood products in one cycle of massive blood transfusion protocol. MTP, massive blood transfusion protocol; BTF, blood transfusion; RBC, red blood cells; FFP, fresh frozen plasma; PLT, platelet. The amount of RBC, FFP, and PLT transfusions is described by Japanese units, which is defined as that derived from 200 mL whole blood.

Our MTP for an obstetric life-threatening hemorrhage provides emergency release of 6 units of RBCs, 3 units of fresh-frozen plasma (FFP), 6 units of RBCs, 4 units of FFP, and 8 units of FFP with 20 units of platelets (PLT) as required (Figure 1). MTP was triggered if any of the following were observed: Shock index > 1.5, or obstetric DIC score > 8 points, or persistent abnormal vital signs. The initial RBC can be compiled and electronically issued in less than 3 min. The entire complement of blood products in the MTP can be delivered to the delivery room or operating room within 60 min. If a patient continues to hemorrhage or future hemorrhage is anticipated after the administration of blood products in the first MTP (MTP-Cycle 1), additional MTP (MTP-Cycle 2) can be activated, which consists of 12, 15, and 20 units of RBC, FFP, and PLT, respectively. While the MTP is in progress, the obstetrician can stop the MTP or change to a normal blood transfusion using a cross-match test if the patient’s condition has been stabilized. However, to prevent accidental errors in such a confusing medical situation, the dosage of blood products cannot be customized on an individual basis.

Antenatal data, such as maternal age, body mass index, parity, mode of conception, number of gestations, and obstetric complications, were collected retrospectively. The diagnosis of hypertensive disorders of pregnancy, gestational diabetes mellitus, and placenta previa was based on the clinical criteria of the Japan Society of Obstetrics and Gynecology [10]. Delivery information, including gestational age at delivery, birth weight, estimated blood loss, mode of delivery, and additional procedures to control bleeding, were also reviewed. We also collected information on MTP, including the etiology of hemorrhage, blood loss, obstetric disseminated intravascular coagulation (DIC) score, and the timing of MTP activation. The amount of RBC, FFP, and PLT transfusions was described by Japanese units, which is defined as that derived from 200 mL whole blood. Amniotic fluid embolism (AFE) was defined based on the Japan consensus criteria for the diagnosis of AFE, which is derived from the United States/United Kingdom criteria [11]. The correlations of the amount of RBC, FFP, and PLT transfusion, blood loss, and obstetrical DIC score [12], which is frequently used in Japan, were analyzed. Furthermore, we collected the following laboratory data: hemoglobin (Hb), platelet count (Plt), prothrombin time (PT), activated partial thromboplastin time (APTT), and fibrinogen (FNG) values at the onset of PPH.

IBM SPSS 25 statistical software (SPSS Inc., Chicago, IL, USA) was used for the statistical analyses. The Mann–Whitney 𝑈 test was used to analyze the continuous variables, and the linear regression analysis was performed to analyze the correlation of the amount of RBC, FFP, and PLT transfusion, obstetrical DIC score, and blood loss. The level of statistical significance was set at *p* < 0.05.

## 3. Results

### 3.1. Patient Characteristics, Obstetric Outcomes, and Intrapartum Management

#### 3.1.1. Patient Characteristic and Obstetric Outcomes

The patient characteristics and obstetric outcomes are shown in Table 1. During the study period, there were 3370 deliveries and 24 obstetrical MTP activations, representing 0.7% of all deliveries. The characteristics of the patients who were treated with MTP are shown in Table 1. The mean maternal age was 36.8 years (range, 31–44), and 20 patients (83.3%) were primiparous. Half of the patients conceived spontaneously, and most (87.5%) were singleton pregnancies. Obstetric complications such as hypertension disorder of pregnancy, gestational diabetes, and placenta previa occurred in approximately 20% of the patients. The mean birth weight of the infants was 2771 g (range, 1709–3532), and 20 infants (83.3%) were delivered by cesarean section.

#### 3.1.2. Intrapartum Management

The data on intrapartum management is presented in Table 2. The mean estimated blood loss was 5017 mL (range, 2200–10,000). The mean obstetric DIC score was 11.7 (range 5–24), and the number of obstetric DIC scores ≥ 8 or ≥13 was 17 (70.8%) and 10 cases (41.7%), respectively. Fourteen patients (58.3%) had a shock index (SI) score ≥ 1. The mean units of RBC, FFP, and PLT given to patients were 17.9 (range, 2–40), 20.2 (range, 3–59), and 20.4 (range, 0–90), respectively. The mean FFP/RBC ratio was 1.13 (range, 0.28–4.00), and the number of FFP/RBC ratio ≥ 1.0, 0.8–1.0, and <0.8 were 16 (66.7%), 3 (12.5%), and 5 (20.8%), respectively. Side effects of blood transfusion occurred in two cases (8.3%), including transfusion-associated circulatory overload and hives. However, acute and delayed hemolytic transfusion reactions were not observed.

Additional procedures to control bleeding were performed in 19 cases, including transarterial embolization (18 cases, 75%), balloon tamponade (2 cases, 8.4%) and hysterectomy (1 case, 4.2%). Admission to the ICU was required in 15 patients (62.5%).

### 3.2. Analysis of the Data in Patients Receiving Blood Products for MTP

#### 3.2.1. Patients Receiving Blood Products for MTP

Table 3 shows the etiologies of the PPH that resulted in MTP activation. The typical causes of PPH treated with MTP included uterine atony (14 cases, 58.3%), AFE (DIC-type) (9 cases, 37.5%), placenta accrete spectrum (PAS; 5 cases, 20.8%), and placenta previa (5 cases, 20.8%). The mean blood loss was 5017 mL (range, 2200–10,000). The mean obstetrical DIC score was 11.7 (range, 5–24). Fourteen patients (58.3%) had an SI ≥ 1. The mean units of RBC, FFP, PLT, and autologous blood transfused to patients were 17.9 (range, 2–40), 20.2 (range, 3–59), 20.4 (range: 0–90), and 1.04 (0–6), respectively. The mean FFP/RBC ratio was 1.13 (range, 0.28–4.00).

#### 3.2.2. The Timing of MTP Activation after Delivery

The variable timing of presentation of PPH was reflected in the timing of MTP transfusion in the postpartum period. Transfusion therapy was commenced <2 h, 2–24 h, and ≥24 h after delivery in 15 (62.5%), 9 (37.5%), and 0 cases (0%), respectively (Table 4).

#### 3.2.3. Correlation of the Amount of RBC, FFP, and PLT Transfusion, Obstetrical DIC Score, and Blood Loss

**Figure 2:**
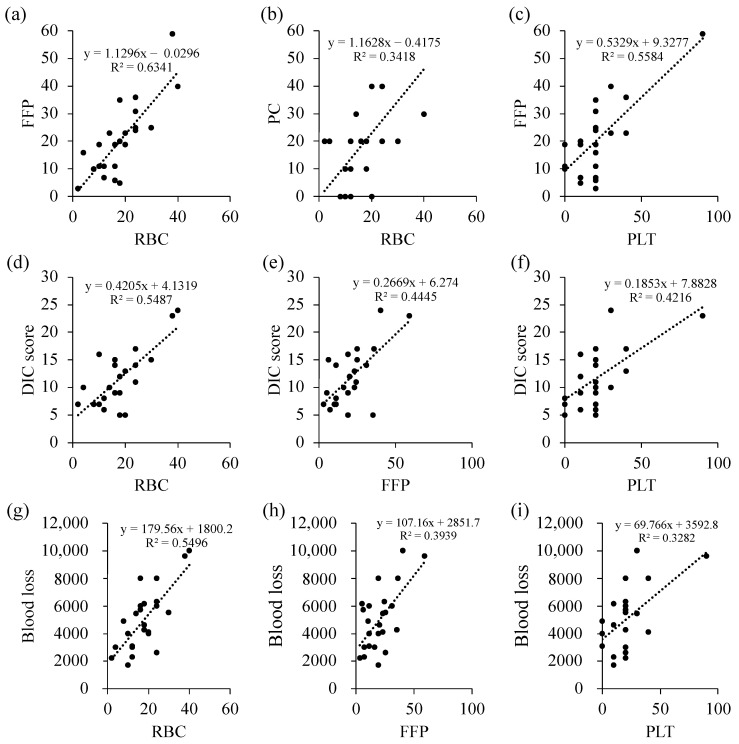
Correlation of the amount of RBC, FFP, and PLT transfusion; obstetrical DIC score; and blood loss. Correlation of RBC and FFP (**a**); RBC and PLT (**b**); FFP and PLT (**c**); DIC score and RBC (**d**), FFP (**e**), and PLT (**f**); blood loss and RBC (**g**); FFP (**h**); and PLT (**i**). DIC, disseminated intravascular coagulation; RBC, red blood cells; FFP, fresh frozen plasma; PLT, platelet. The amount of RBC, FFP, and PLT transfusions is described by Japanese units, which is defined as that derived from 200 mL whole blood.

**Figure 3:**
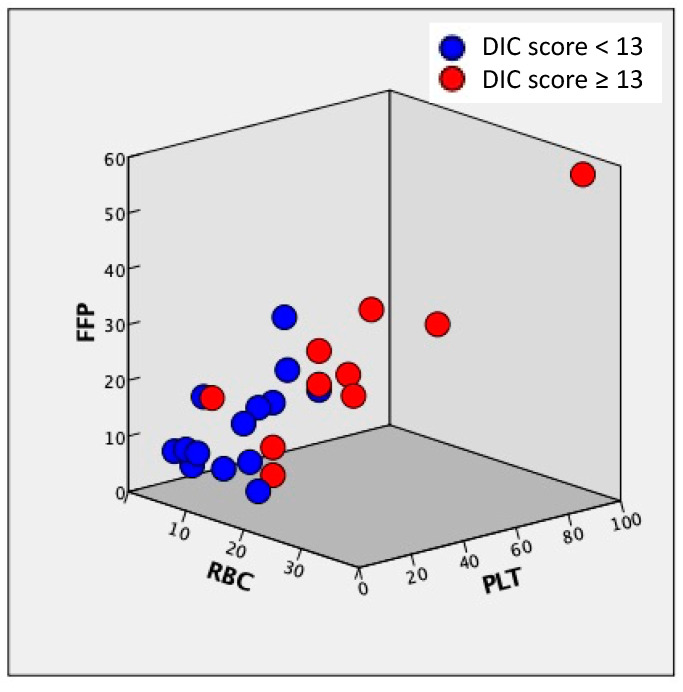
Correlation of RBC, FFP, and PLT. RBC, red blood cells; FFP, fresh frozen plasma; PLT, platelet. The amount of RBC, FFP, and PLT transfusions is described by Japanese units, which is defined as that derived from 200 mL whole blood.

We observed a correlation between the amount of RBC, FFP, and PLT transfusion, obstetrical DIC score, and blood loss (Figure 2, Table 5). The correlation coefficients of any two items among RBC, FFP, PLT, blood loss, and DIC ranged from 0.757 to 0.892 (Table 5), indicating high levels of correlation coefficients. Figure 3 shows the correlation of RBC, FFP, and PLT, and the relationship between the amount of RBC, FFP, and PLT transfusion is plotted (Figure 3). The ratio of RBC, FFP, and PLT doses was approximately 1:1:1, and the total amount of blood transfusion increased in patients with obstetrical DIC score ≥ 13 compared with those with <13 (Figure 3).

#### 3.2.4. Clinical and Laboratory Parameters at the Onset of PPH in Patients Who Received MTP

The clinical and laboratory parameters at the onset of PPH in patients who received MTP are shown in Table 6. The mean Hb, Plt, FNG, APTT, and PT-INR were 6.5 (range, 4.1–9.1), 11.9 (range, 6.8–22.1), 150.1 (range, <50–332), 51.5 (range, 27.1–125.7), and 1.34 (range, 0.94–2.82), respectively. In case 14, the laboratory parameters at the onset of PPH were not determined because emergency cesarean section and MTP activation were concurrently performed following the clinical diagnosis of uterine rupture.

**Figure 4:**
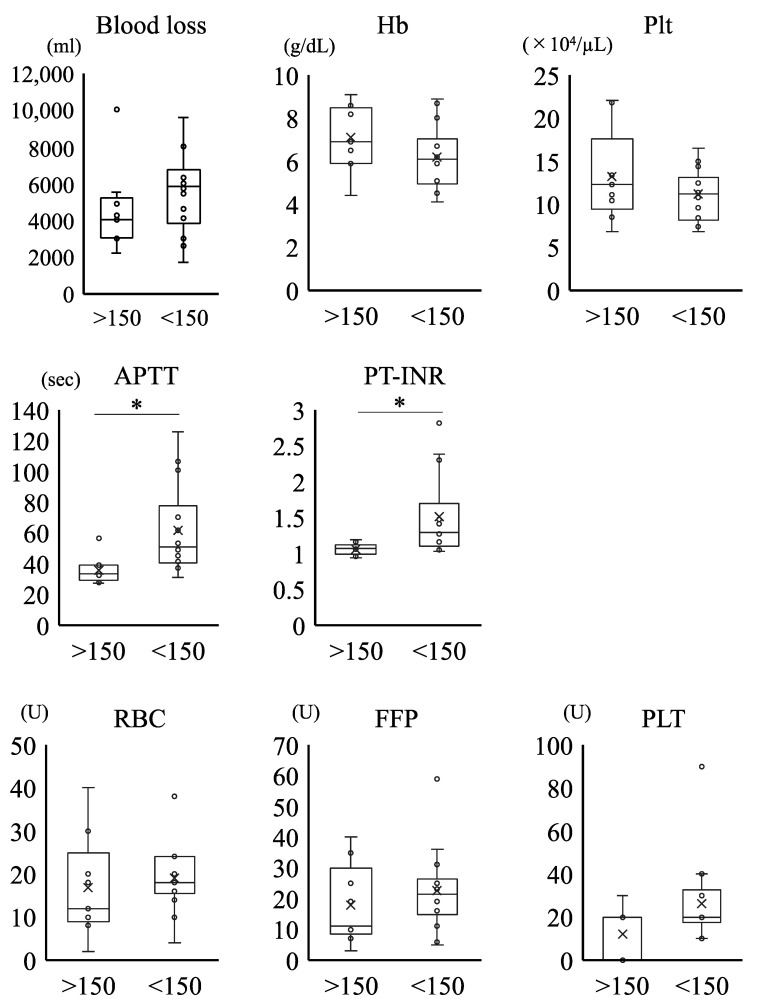
Comparison of clinical and laboratory parameters between ≥ or <150 mg/dL fibrinogen (FNG) groups. Hb, hemoglobin concentration (normal range, 11.6–14.8 g/dL); Plt, platelet count (normal range, 15.8–34.8 104/μL); FNG, fibrinogen (normal range, 150–400 mg/dL); APTT, activated partial thromboplastin time (normal range, 24–39 s); PT-INR, International Normalized Ratio of Prothrombin Time (normal range, 0.90–1.10). The amount of RBC, FFP, and PLT transfusions is described by Japanese units, which is defined as that derived from 200 mL whole blood. *: *p* < 0.05.

Figure 4 shows the comparison of the clinical and laboratory parameters between the groups with FNG levels ≥ 150 mg/dL and <150 mg/dL. We found that the APTT and PT-INR levels were significantly higher in the <150 mg/dL FNG group than in the ≥150 mg/dL FNG group. Conversely, the amount of blood loss and blood transfusion (RBC, FFP, and PLT) and the Hb and Plt levels were comparable between the two groups.

We compared the clinical and laboratory parameters between FNG groups ≥150 or <150 mg/dL. The APTT and PT-INR levels were significantly higher in the < 150 mg/dL FNG group than in the ≥150 mg/dL FNG group. Conversely, the amount of blood loss and blood transfusion (RBC, FFP, and PLT) and the Hb and Plt levels were comparable between the two groups.

## 4. Discussion

In this study, we found that the incidence of PPH with MTP was 24 per 3370 deliveries (0.7%). The typical causes of PPH treated with MTP included uterine atony (14 cases, 58.3%), AFE (DIC-type) (9 cases, 37.5%), PAS (5 cases, 20.8%), and placenta previa (5 cases, 20.8%). Our MTP could provide a large amount of blood transfusion in a short period. Specifically, the mean units of RBC, FFP, and PLT transfused to patients were 17.9, 20.2, and 20.4 units, respectively. The FFP/RBC ratio was maintained above 1.0, and there was a correlation between the amount of RBC, FFP, and PLT transfusion, with a ratio of approximately 1:1:1. Thus, our MTP could contribute to improving maternal outcomes in patients with severe PPH.

We found that our institutional rate of MTP activation was 0.7%, which was about ten times that of previous reports (2.3–9.1 per 10,000 deliveries) [7,13,14,15]. This may be because our hospital is a university hospital and handles many high-risk cases. Consistent with previous reports [7,13,14,15], most of the MTP therapy was commenced within 2 h after delivery, and the indications for MTP activation were heterogeneous. Although the specific risk factors of women requiring MTP after delivery have not been determined and have only been addressed in prior studies [1,7,13], the common etiologies of obstetric hemorrhage reported in our study were partially consistent with data from previous population-wide studies of PPH (uterine atony, PAS, and placenta previa) [1,7,13]. In Japan, AFE can be categorized into the following based on the initial symptoms: (i) AFE that starts with cardiopulmonary collapse and is characterized by pulmonary/respiratory symptoms, and (ii) AFE that starts with atonic bleeding/DIC (DIC-type AFE) [11]. All the included AFE cases we encountered at our institution were categorized the DIC-type AFE, which is a disease that causes massive bleeding from the uterus due to severe uterine atony. Considering the differences in the diagnostic criteria of AFE among countries, the common etiologies of obstetric hemorrhage include severe cases of uterine atony refractory to various treatments, which are defined as DIC-type AFE in Japan. Recent evidence suggests that the rate of uterine atony is increasing worldwide, especially in developed countries [7,13,14,15]. Therefore, obstetricians must be prepared for the possibility of unanticipated massive atonic bleeding associated with DIC, especially within 2 h after delivery.

In this study, we found that the obstetrical DIC score, which indicates the severity of DIC, was also high in the patients treated with MTP. A recent analysis by Japan’s Maternal Death Exploratory Committee and the Amniotic Fluid Embolism Registry states that “there is concern that AFE may be involved in massive obstetric hemorrhage,” and the DIC-type AFE is characterized by the sudden onset of DIC associated with severe obstetric hemorrhage [11]. In patients with severe PPH, a previous report indicated that coagulation factors such as FNG, factor V, antithrombin, and protein C decreased, while PT and thrombin/antithrombin increased in the early phase of severe PPH [16]. High levels of obstetric DIC score and laboratory data showed that there was a marked decrease in FNG and Plt, and the increase in PT-INR and APTT in our study was consistent with these findings (Table 5).

Among the abnormalities of the coagulation system in the early stages of critical PPH after delivery, low FNG levels may be the characteristic feature of PPH [17]. A previous report indicated that the FNG level was lower in cases of severe hemorrhage, and the decrease in FNG level is an early predictor of the severity of PPH [16]. Significant disturbances in the APTT and PT levels were observed in patients with low FNG levels (Figure 4). To overcome this problem, FFP dissolution should be initiated using the two thawers in the hospital as soon as the MTP is activated, allowing us to utilize small and easy to dissolve units, including three units of FFP in 15 min, four units in 40 min, and eight units in 60 min, for a total of 15 units of FFP within an hour (Figure 1b). Moreover, our protocol was designed to enable initial RBC transfusion in a short period to prevent dilutional coagulopathy due to excessive fluid replacement (Figure 1b). This study clarified the effectiveness of our MTP, especially with regard to early coagulation factor replacement and prevention of dilutional coagulopathy, on severe PPH, as highlighted by our data that showed that the total amount of blood loss and blood transfusions were comparable between the two groups distinguished by FNG levels at the onset of PPH (Figure 4). Previously, Rahe-Meyer et al. reported that FNG concentrates enabled a more rapid and effective control of intraoperative bleeding when compared with transfusion of four units of FFP and two units of platelets [18]. Similarly, the results of a national survey of FNG concentrate usage for PPH in Japan indicated its usefulness for massive PPH. In this study, we used only FFP to provide coagulation factor replacement without the use of an FNG concentrate [19]. In addition, we have not used adjuncts such as tranexamic acid, calcium, or more fibrinogen-focused components such as a cryoprecipitate in our MTP. Given that DIC and low fibrinogen were identified as major risk factors for increased volume of blood administered, we want to investigate the effect of these adjuncts in our MTP in further study.

In MTP, optimization of the ratio of RBC to FFP administration must be considered to prevent dilutional coagulopathy. In our protocol, the ratio of RBC to FFP administration was 4: 5 (Figure 1b). Blood products are typically administered rapidly before the patient’s condition stabilizes. In contrast, we slowly replenished the insufficient amount of blood products after the patient’s condition had stabilized. The results showed that there was a correlation between the amount of RBC, FFP, and PLT transfusion, and the ratio was approximately 1:1:1 (Figure 2 and Figure 3, Table 5). To date, most studies of MTP were based on trauma or military care, and a previous report suggested that among the patients receiving MTP, only about 2% were related to obstetric hemorrhage [20]. In the field of trauma, a high FFP/RBC ratio (1:1) has been reported to improve outcomes and survival in patients with massive bleeding [9,21,22]. In obstetrics, the early administration of MTP and a high FFP/RBC ratio over 1:1 have been recommended for massive PPH [23]. As shown in this study, the MTP in our institution was proven to be successful, making it possible to transfuse a large amount of blood in a short period, while maintaining an appropriate RBC to FFP ratio. However, it is necessary to verify whether the protocol is applicable to other facilities in future studies.

In Japan, an electronic system is required to order blood transfusion in the hospital. Although this electronic ordering system is accurate, it is complicated to order. This is most problematic in situations where there are few obstetricians on holidays and at night, as many hospitals in Japan. Since an incorrect transfusion order can lead to serious complications for the patient, it is necessary to ensure that the electronic order is accurate, including in situations of emergency such as a massive PPH. We believe that our MTP is an innovative system that enables all transfusion orders to be done accurately with one click in the electronic system, and we also believe that our MTP could contribute to a more stable 24 h service for obstetric hemorrhage in our hospital.

## 5. Conclusions

The management of severe PPH after delivery is a challenging task for obstetric and hospital transfusion teams. Our MTP provides early access to blood products for patients experiencing severe PPH, contributing to improved maternal outcomes after resuscitation in our hospital. A hospital-based MTP specific to the obstetric field can provide life-saving transfusion therapy for patients presenting with severe PPH. In this study, the diverse etiology of PPH, high volumes of reported blood loss, high rate of blood product usage, and early coagulation factor loss suggest that an MTP may be an important therapeutic measure for managing unanticipated bleeding after delivery. We believe that it is necessary to verify through future studies whether the addition of more fibrinogen-focused components such as FNG concentrate to our MTP is effective in our hospital and if MTP is applicable to other facilities. The MTP should be optimized according to the characteristics of each facility, such as the amount of transfusion reserves, the number of FFP dissolution equipment, and manpower. Our study suggests the implementation of a hospital-specific MTP protocol to improve the supply and utilization of blood products to physicians managing major obstetric hemorrhages.

## Figures and Tables

**Table 1:** Patient characteristics and obstetric outcomes.

Maternal age (years)	36.8 (31–44)
Body mass index (kg/m^2^)	21.0 (15.4–32.4)
Primiparous	20 (83.3)
Spontaneous pregnancy	12 (50.0)
Singleton pregnancy	21 (87.5)
Obstetric complication	
Hypertension disorder of pregnancy	4 (16.7)
Gestational diabetes	6 (25.0)
Placenta previa	5 (20.8)
Gestational age at delivery (weeks)	37.4 (33.3–40.7)
Birth weight (g)	2771 (1709–3572)
Mode of delivery	
Cesarean section	20 (83.3)
Spontaneous vaginal delivery	2 (8.3)
Instrumental delivery	2 (8.3)

Data are shown as mean (range) or number (%).

**Table 2:** Intrapartum management.

Estimated blood loss (mL)	5017 (2200–10,000)
Obstetric DIC score	11.7 (5–24)
≥8	17 (70.8)
≥13	10 (41.7)
SI ≥ 1	14 (58.3)
Blood transfusion	
RBC (U)	17.9 (2–40)
FFP (U)	20.2 (3–59)
PLT (U)	20.4 (0–90)
FFP/RBC ratio (mean)	1.13
≥1.0	16 (66.7)
0.8–1.0	3 (12.5)
<0.8	5 (20.8)
Additional procedure to control bleeding	
Transarterial embolization	18 (75.0)
Balloon tamponade	2 (8.4)
Hysterectomy	1 (4.2)
ICU admission	15 (62.5)

Data are shown as mean (range) or number (%). The amount of RBC, FFP, and PLT transfusions is described by Japanese units, which is defined as that derived from 200 mL whole blood.

**Table 3:** List of patients receiving blood products for massive transfusion protocol.

No.	Etiology of Hemorrhage	Blood Loss (mL)	Obstetrics DIC Score	SI ≥ 1	RBC (U)	FFP (U)	PLT (U)	Autologous Blood (U)	FFP/RBC Ratio
1	AFE (DIC-type), Uterine atony	8000	17	+	24	36	40	0	1.5
2	AFE (DIC-type), Uterine atony	2600	17	+	24	25	20	0	1.04
3	Uterine atony	10,000	24	+	40	40	30	0	1.00
4	Placenta previa, PAS	5500	15	+	30	25	20	2	0.83
5	PAS	4900	7	+	8	10	0	0	1.25
6	Placenta previa, Uterine atony	8000	9	+	16	19	20	4	1.19
7	AFE (DIC-type), Uterine atony	5700	15	+	16	6	20	0	0.38
8	PAS	3000	10	−	4	16	20	6	4.00
9	AFE (DIC-type), Uterine atony	4100	13	+	20	23	40	0	1.20
10	Vaginal hematoma	6000	14	−	16	11	20	0	0.69
11	Uterine atony	2200	7	−	2	3	20	0	1.50
12	Uterine atony	4000	7	−	10	11	0	0	1.10
13	AFE (DIC-type), Uterine atony	4600	12	−	18	20	10	0	1.11
14	Uterine rupture	2300	6	−	12	7	10	0	0.58
15	Placenta previa, HELLP syndrome	9600	23	+	38	59	90	3	1.55
16	AFE (DIC-type), Uterine atony	6000	14	+	24	31	20	0	1.29
17	Vaginal hematoma	4000	5	−	20	19	0	0	0.95
18	AFE (DIC-type), Uterine atony, Placenta previa, PAS	6150	9	+	18	5	10	6	0.28
19	AFE (DIC-type), Placental abruption	6300	11	+	24	24	20	0	1.00
20	Uterine atony, Placenta previa	5433	10	−	14	23	30	4	1.64
21	Uterine atony	3000	6	−	12	7	20	0	0.58
22	AFE (DIC-type)	1700	16	+	10	19	10	0	1.90
23	PAS	4240	5	−	18	35	20	0	1.94
24	Uterine atony	3090	8	+	12	11	0	0	0.92
	mean or number (range or %)	5017	11.7	14	17.9	20.2	20.4	1.04	1.13
		(2200–10,000)	(5–24)	(58.3)	(2–40)	(3–59)	(0–90)	(0–6)	(0.28–4.00)

Data are shown as mean (range) or number (%). AFE, amniotic fluid embolism; DIC, disseminated intravascular coagulation; PAS, placenta accreta spectrum; SI, shock index; MTP, massive transfusion protocol; RBC, red blood cells; FFP, fresh frozen plasma; PLT, platelet; +, Yes, −, No. The amount of RBC, FFP, and PLT transfusions is described by Japanese units, which is defined as that derived from 200 mL whole blood.

**Table 4:** The timing of MTP activation after delivery.

<2 h	15 (62.5%)
2–24 h	9 (37.5%)
>24 h	0 (0%)

**Table 5:** Correlation coefficient of RBC, FFP, PLT, obstetrical DIC score, and blood loss.

	RBC	FFP	PLT	Obstetrical DIC Score	Blood Loss
RBC	1.000	-	-	-	-
FFP	0.892	1.000	-	-	-
PLT	0.765	0.864	1.000	-	-
Obstetrical DIC score	0.861	0.817	0.806	1.000	-
Blood loss	0.861	0.792	0.757	0.774	1.000

DIC, disseminated intravascular coagulation; RBC, red blood cells; FFP, fresh frozen plasma; PLT, platelets. The amount of RBC, FFP, and PLT transfusions is described by Japanese units, which is defined as that derived from 200 mL whole blood.

**Table 6:** Clinical and laboratory parameters at the onset of PPH in patients who received MTP.

No.	Etiology of Hemorrhage	Blood Loss (mL)	Hb (g/dL)	Plt (×10^4^/μL)	FNG (mg/dL)	APTT (s)	PT-INR
1	AFE (DIC-type), Uterine atony	8000	6.2	7.4	<50	125.7	2.82
2	AFE (DIC-type), Uterine atony	2600	6.7	12.7	<50	55.7	2.38
3	Uterine atony	10,000	4.4	8.5	173	38.9	1.08
4	Placenta previa, PAS	5500	6.9	6.8	203	27.1	0.96
5	PAS	4900	8.2	10.4	172	30.3	1.08
6	Placenta previa, Uterine atony	8000	4.5	10.8	94	53.1	1.3
7	AFE (DIC-type), Uterine atony	5700	6	15	146	37.3	1.05
8	PAS	3000	8.9	12.5	136	45.3	1.16
9	AFE (DIC-type), Uterine atony	4100	5.9	16.5	147	41.6	1.1
10	Vaginal hematoma	6000	4.5	8.4	144	101.1	1.41
11	Uterine atony	2200	8.6	12.6	267	35.2	1.01
12	Uterine atony	4000	5.9	12.3	177	56.6	1.07
13	AFE (DIC-type), Uterine atony	4600	5.1	6.8	91	70.1	1.27
14	Uterine rupture	2300	ND	ND	ND	ND	ND
15	Placenta previa, HELLP syndrome	9600	4.1	14.4	118	48.9	1.29
16	AFE (DIC-type), Uterine atony	6000	6.4	12.5	131	38	1.09
17	Vaginal hematoma	4000	9.1	22.1	332	27.7	0.94
18	AFE (DIC-type), Uterine atony, Placenta previa, PAS	6150	8.7	7.4	64	61.8	1.48
19	AFE (DIC-type), Placental abruption	6300	6.4	9.6	72	47.3	1.49
20	Uterine atony, Placenta previa	5433	5.2	11.4	137	31.1	1.03
21	Uterine atony	3000	8.4	21.8	328	33.7	1.16
22	AFE (DIC-type)	1700	8	11	<50	106.4	2.3
23	PAS	4240	6.5	11.1	153	32.7	1.19
24	Uterine atony	3090	5.9	13.4	218	39.1	1.06
	mean (range)	5017 (2200–10,000)	6.5 (4.1–9.1)	11.9 (6.8–22.1)	150.1 (<50–332)	51.5 (27.1–125.7)	1.34 (0.94–2.82)

Data are shown as mean (range) or number (%). AFE, amniotic fluid embolism; DIC, disseminated intravascular coagulation; PAS, placenta accreta spectrum; Hb, hemoglobin concentration (normal range, 11.6–14.8 g/dL); Plt, platelet count (normal range, 15.8–34.8 104/μL); FNG, fibrinogen (normal range, 150–400 mg/dL); APTT, activated partial thromboplastin time (normal range, 24–39 s); PT-INR, international normalized ratio of prothrombin time (normal range, 0.90–1.10); ND, not determined.

## Data Availability

The data presented in this study are available on reasonable request from the corresponding author.
